# Adverse health events and recommended health research priorities in agility dogs as reported by dog owners

**DOI:** 10.3389/fvets.2023.1127632

**Published:** 2023-03-13

**Authors:** Debra C. Sellon, Denis J. Marcellin-Little, Dianne McFarlane, Molly McCue, Arielle Pechette Markley, Abigail Shoben

**Affiliations:** ^1^Department of Veterinary Clinical Sciences, College of Veterinary Medicine, Washington State University, Pullman, WA, United States; ^2^Department of Surgical and Radiological Sciences, School of Veterinary Medicine, University of California, Davis, Davis, CA, United States; ^3^Department of Large Animal Clinical Sciences, College of Veterinary Medicine, University of Florida, Gainesville, FL, United States; ^4^Department of Veterinary Population Medicine, College of Veterinary Medicine, University of Minnesota, Minneapolis, MN, United States; ^5^Department of Veterinary Clinical Sciences, College of Veterinary Medicine, The Ohio State University, Columbus, OH, United States; ^6^Division of Biostatistics, College of Public Health, The Ohio State University, Columbus, OH, United States

**Keywords:** agility, dog, sports medicine, research priorities, orthopedic injuries, dog owners, infectious diseases

## Abstract

**Objective:**

To understand relative frequency of adverse health events, defined as injuries or infectious diseases, in dogs participating in agility and to determine health research priorities of agility dog owners.

**Procedures:**

An internet-based questionnaire distributed to agility dog owners included items related to experiences with infectious diseases and injuries in agility dogs, reasons for retirement of dogs from competition, and ranking of health research priorities. Frequencies of infectious diseases in US geographic regions were compared with Chi-square tests. Research priority rankings were determined as median and interquartile range (IQR) for each topic. Rank-based tests (Kruskal Wallis and Mann-Whitney) compared rankings between participants in different agility organizations, between veterinarian and non-veterinarian competitors, and between respondents who had competed in national championship events and other respondents.

**Results:**

There were 1,322 respondents who had competed in canine agility in the previous 6 months, with those respondents reporting a median time competing in the sport of 13 years (IQR = 8–20 years); 50% of respondents had competed in at least one national championship agility event in the preceding 5 years. Overall, 1,015 respondents (77%) indicated that one or more of their dogs had been injured and approximately one-third (*n* = 477, 36%) indicated that one or more dogs had likely acquired one or more infectious diseases as a result of agility activities. Specific types of infectious diseases acquired varied by geographic region in the US. Research priority rankings were similar regardless of preferred agility organization or respondent experience. The highest-ranking research topics were identification of risk factors for specific types of injuries, improvements in equipment and understanding of safe course design, and physical conditioning programs to prevent injury.

**Conclusions and clinical relevance:**

Agility competitors prioritize research in areas that advance understanding of injury prevention in their dogs. Research priorities are nearly uniform among competitors regardless of their preferred agility organization or level of experience, providing a strong rationale for agility organizations to collaborate in research initiatives that improve safety and well-being for dogs competing in the sport. There has been little published research focusing on the high-priority research areas identified by competitors.

## Introduction

Agility has grown in popularity and is now one of the most popular dog sports in North America (1). The number of scientific studies focused on agility dog health, exercise science, training, and behavior is increasing (2–19). Recent publications have primarily focused on identification of risk factors for injuries in agility dogs (7–9, 13, 14, 18–20) and kinematics of obstacle performance (2, 3, 5, 6, 12, 16, 17). Almost all of the publications related to types of injuries incurred by agility dogs and risk factors for those injuries are based on data provided by agility dog handlers through internet-based questionnaires rather than from review of veterinary medical records (9, 13–15, 19–25). This type of data is useful but may be affected by distribution, selection, and response biases (26, 27). Kinematic analyses have investigated performance of specific obstacles to understand movement patterns (2, 3, 5, 6, 12, 16), correlate movement patterns with obstacle performance (6), and evaluate differences between novice and experienced dogs (17). These kinematic studies are generally not correlated or analyzed with the primary goal of understanding injuries or injury prevention.

The number of organizations which sponsor agility competitions is increasing as the sport increases in popularity. The types of agility courses that are designed for competition can vary widely between organizations with regards to technical requirements such as types of obstacles, numbers of obstacles, and obstacle spacing. One consequence of the growing number of agility enthusiasts, is the increased willingness of participants to travel long distances to participate in major regional or national events. Each sponsoring organization may have several such events each year. This results in dogs that are traveling long distances and commingling in shared air spaces with minimal attention to biosecurity practices. The possibility of infectious disease outbreaks among dogs participating in canine sporting events is recognized by the American Kennel Club (AKC) and its Canine Health Foundation sponsored development of guidelines for disease prevention in canine group settings (28). Despite recognition of disease risk by AKC, the largest sponsoring organization for canine agility in the United States, AKC agility trials have almost no disease prevention requirements and there have been no investigations of disease associated with agility events.

Organizations that sponsor agility have no formal association or governing body and there is little apparent communication between these groups. This fragmented sport governance structure makes it challenging to combine resources to develop comprehensive information related to injuries or disease associated with the sport of agility or to pursue larger-scale research initiatives that may advance the health and safety of dogs participating in all forms of agility. There are similar challenges within the much larger equine sport industry with multiple types of equine sports and associated governing bodies. These groups have been brought together on occasion, however, to pursue research initiatives or disease control programs of importance to all horses.

Health priority surveys have provided evidence to justify and encourage collaboration among groups and identify the most important health concerns of participants. These efforts have led to increased research funding opportunities through collaboration of multiple equine industry groups and veterinary funding organizations. Similarly, in human medicine, processes that include physicians, researchers, and patients have been used to broaden perspectives and identify gaps in evidence with a goal of setting research priorities (29–32). A similar approach might be used to increase funding and support for research which advances the health and well-being of agility dogs. The first step in this process requires identification of common health concerns and research priorities of participants and organizations.

We hypothesized that the injury and disease experiences and perceptions of research priorities among agility handlers would be similar regardless of their preferred agility organization. The aims of the research reported here were to obtain information from agility dog owners regarding their experiences with adverse health events associated with participation in agility activities and to determine their perception of research priorities. This information is important to guide communication with agility organizations and canine health funding groups to ensure that agility dog health needs are appropriately identified and adequately investigated.

## Materials and methods

### Questionnaires

An internet-based questionnaire for dog owners was designed on a commercial internet survey site (Qualtrics, Provo, UT, www.qualtrics.com). This questionnaire consisted of 24 items separated into 5 sections: introduction, experiences with health issues in agility dogs, research priorities, sources of agility dog health information, and dog owner background and experiences. The full text of the questionnaire is available as Supplementary Item 1. Data from the section on sources of agility dog health information are not included in this report. For inclusion in the final data set, respondents were required to be > 18 years of age, reside in the United States (US), and have competed in at least one agility trial in the previous 6 months.

To develop the questionnaire, a draft was prepared based on the experiences and expertise of the authors and distributed to a small number of individuals who were involved in dog agility. These individuals included veterinary professionals and non-veterinary agility competitors. These individuals provided information related to required time for completion and clarity and completeness of content. Based on this feedback, minor modifications were made. Test responses were deleted from the software before distribution of the final questionnaire.

Respondent experiences with adverse health issues in agility dogs were assessed by asking each individual to indicate whether they had ever had a dog that had experienced an injury to specific areas of the body. Only injuries that caused the respondent to moderate or stop agility training or competition with the injured dog were included. Respondents also indicated whether any of their personal dogs had acquired any of a wide range of infectious diseases as a result of interactions with other dogs at agility training or competition events and to indicate the reason why they had discontinued or retired an agility dog from competition (most recent dog retired). After describing these personal experiences, respondents ranked 12 broad areas of research related to agility dog health and safety from most important (1) to least important (12). Respondents were also asked to select up to three topics of highest priority (unranked) for musculoskeletal injury research related to agility dogs.

The experience of respondents with dogs and within the sport of canine agility was assessed with questions relating to the number of dogs owned, years involved in agility activities, total days per year of competition, breeding of dogs, agility organizations in which the respondent had competed in the past 5 years, designation of the agility organization with which the individual had most frequently competed, attendance at a national championship event within the past 5 years, and the types of agility-related activities in which the individual was engaged. Respondents were asked to indicate the region of the US in which they resided by providing the first digit of the zip code of their primary residence.

Distribution of the questionnaire was initiated on 30 November 2021 and data collection was terminated on 30 January 2022. Invitations to respond to the questionnaire were distributed by all authors through social media sites and dog organizations that were relevant to agility enthusiasts. The questionnaire was accessed by clicking on a hyperlink in the message.

The Institutional Review Board of Washington State University determined this project satisfied the criteria for exempt research. All survey responses were anonymous and datasets generated and/or analyzed for this report are available upon reasonable request to the authors.

### Data analysis

Statistical analyses were performed using commercial statistical software (SigmaStat 4.0, Systat Software, Inpixon, Palo Alto and Stata 15.1, StataCorp, College Station) with significance set at *P* < 0.05 unless specified otherwise. Responses to each item were summarized individually in tabular form based on normality of data as mean with standard deviation, median with 25th and 75th quartiles, and 95% confidence intervals (CI).

Using a conservative estimated population size of 1,000,000 individuals in the United States who might be competing in agility (true numbers are not available), a sample size of 1,066 was needed to estimate 95% confidence intervals for proportions with a 3% margin of error (33).

The geographic distribution of the respondents was assessed by calculating the number of responses from each region of the US, based on the first digit of the zip code provided by respondents. Total number of respondents in each region and the response rate per million population in each region, based on 2010 US census data, were calculated.

Respondents indicated whether they believed any dog they owned had acquired any of 11 possible infectious diseases as a result of interactions with other dogs at agility training or competition events. Responses were curated into four groups consisting of infectious respiratory disease (cough or respiratory disease of unknown type, canine influenza, or kennel cough [infectious tracheobronchitis]), external parasitic diseases (fleas, ticks, or similar external parasites), gastrointestinal diseases (intestinal parasites of any type, diarrhea or vomiting of unknown type, canine parvovirus), or other infectious diseases (coronavirus, distemper, hepatitis virus, leptospirosis, other specified disease). Incidence of these groups of infectious diseases in each geographic region was compared by Chi-square analysis. When *P* < 0.05, individual relationships were explored with Chi-square analysis using West Coast respondents (zip prefix = 9) as the reference group.

The median and interquartile range (IQR) for the ranking of each potential area of research were calculated for the total respondent population. Rankings for individual items were compared between participants in different agility organizations using Kruskal-Wallis tests. Mann-Whitney rank sum tests were used to compare rankings for individual items between respondents who were veterinarians or credentialed veterinary technicians and respondents who were not veterinary professionals. A similar comparison was made between respondents who had competed in national championship events and those who had never competed in national championship events. Within each comparison group (12 tests), the Holm correction for multiple comparisons was used to maintain the overall false positive rate at 0.05.

## Results

### Questionnaire responses

A total of 2,215 respondents accessed the questionnaire. Of these, 893 respondents were eliminated because 680 respondents did not complete the questionnaire, 5 respondents were < 18 years of age, 47 respondents did not reside in the US or did not provide the first digit of their zip code, and 161 respondents had not competed in agility in the 6 months preceding their response to the survey. The final data set included responses from 1,322 individuals who met all inclusion criteria.

### Respondent characteristics and experiences

The geographic distribution of respondents is shown in Table 1. The mean response rate per million inhabitants for all zip code regions, calculated using data from the 2010 census, was 4.6 ± 2.0 per million population, with a range of 2.2–8.7 per million inhabitants for various regions.

**Table 1:** Respondent numbers from regions of the US as defined by first digit of zip code for each respondent's primary residence.

**First digit of zip code**	**States and territories**	**Number of responses**	**Zip code population (millions)**	**Response rate per million**
0	Connecticut, Massachusetts, Maine, New Hampshire, New Jersey, Puerto Rico, Rhode Island, Vermont, Virgin Islands, Army Post Office Europe, Fleet Post Office Europe	142	23.2	6.1
1	Delaware, New York, Pennsylvania	90	33.0	2.7
2	District of Columbia, Maryland, North Carolina, South Carolina, Virginia, West Virginia	114	30.4	3.8
3	Alabama, Florida, Georgia, Mississippi, Tennessee, Army Post Office Americas, Fleet Post Office Americas	103	42.6	2.4
4	Indiana, Kentucky, Michigan, Ohio	121	32.2	3.8
5	Iowa, Minnesota, Montana, North Dakota, South Dakota, Wisconsin	144	16.6	8.7
6	Illinois, Kansas, Missouri, Nebraska	128	23.5	5.4
7	Arkansas, Louisiana, Oklahoma, Texas	81	36.3	2.2
8	Arizona, Colorado, Idaho, New Mexico, Nevada, Utah, Wyoming	112	21.1	5.3
9	Alaska, American Samoa, California, Guam, Hawaii, Marshall Islands, Federated States of Micronesia, Northern Mariana Islands, Oregon, Palau, Washington, Army Post Office Pacific, Fleet Post Office Pacific	287	49.9	5.8
	Total, all regions	1,322	308.8	4.3

The total population for each zip code was obtained from 2010 census data: https://blog.splitwise.com/2013/09/18/the-2010-us-census-population-by-zip-code-totally-free/.

Of the 1,319 respondents who specified the number of dogs they owned that were currently competing in agility, most respondents were currently competing with either one (*n* = 591, 44.8%) or two (*n* = 499, 37.8%) dogs, with 155 handlers (11.7%) competing with 3 dogs, and 60 handlers (4.5%) reporting currently competing with 4 or more dogs. The median lifetime number of dogs with which respondents had competed was 4 dogs (interquartile range [IQR] = 3 to 6). Median number of years competing in agility was 13 years (IQR = 8 to 20). Median total days/year competing in agility was 31 days (IQR = 20 to 50). There were 299 respondents (22.7%) who indicated that they had been involved with dog breeding in the previous 10 years as the owner of either the dam or sire of a litter of puppies. Almost half of the respondents (*n* = 658, 49.8%) had competed in one or more national championship agility event in the past 5 years. The number of respondents participating in events sponsored by each of the major US agility organizations and the number of respondents indicating each organization as their most frequent competition venue are shown in Table 2. Respondents' roles and types of involvement within the sport of agility are summarized in Table 3.

**Table 2:** Number (percent) of 1,322 respondents who indicated that they had competed in agility competitions sponsored by each major agility organization in the past 5 years and number (percent) of 1,319 respondents who indicated that the specified organization had sponsored the competitions in which they had competed most frequently in the past 5 years.

**Agility organization**	**Competed in organization sponsored events in last 5 years**	**Most frequent competition organization in past 5 years**
American Kennel Club (AKC)	1130 (85.5%)	737 (55.9%)
Canine Performance Events (CPE)	466 (35.2%)	203 (15.4%)
United States Dog Agility Association (USDAA)	609 (46.1%)	184 (13.9%)
North American Dog Agility Council (NADAC)	321 (24.3%)	69 (5.2%)
United Kingdom International Agility (UKI)	529 (40.0%)	68 (5.2%)
Australian Shepherd Club of America (ASCA)	278 (21.0%)	43 (3.3%)
Teacup Dog Agility Association (TDAA)	72 (5.4%)	10 (0.8%)
United Kennel Club (UKC)	57 (4.3%)	3 (0.2%)
Dogs on Course in North America (DOCNA)	17 (1.3%)	0 (0.0%)

**Table 3:** Number (%) of 1,321 respondents indicating the specified type of participation in canine agility.

**Which of the following best describe your current participation in the sport of dog agility?**	**Number (%) of respondents**
Active competitor and trainer with my personal dogs	1272 (96.3%)
Instructor for other agility handlers and their dogs (paid for services)	252 (19.1%)
Dog breeder	117 (8.9%)
Agility trial secretary or similar activity	115 (8.7%)
Owner or manager of an agility or dog-related retail business	73 (5.5%)
Training a dog but not currently competing	69 (5.2%)
Currently practicing or retired veterinarian	69 (5.2%)
Agility judge	65 (4.9%)
Active competitor with dogs owned by other individuals	57 (4.3%)
Other, please specify^*^	49 (3.7%)
Owner or manager of a dog sports venue	35 (2.6%)
Member of the leadership group of an agility organization such as AKC or NADAC	34 (2.6%)
Currently practicing or retired credentialed veterinary technician	25 (1.9%)

Respondents were allowed to select multiple responses. ^*^Specified roles included 4-H leader or youth instructor, unpaid agility instructor, club members who assisted with agility trials, canine massage therapist, trigger point therapist, canine physical therapist, canine chiropractor, human physical therapist, sport psychologist, and veterinary office manager.

### Agility-related injuries and illnesses

**Figure 1:**
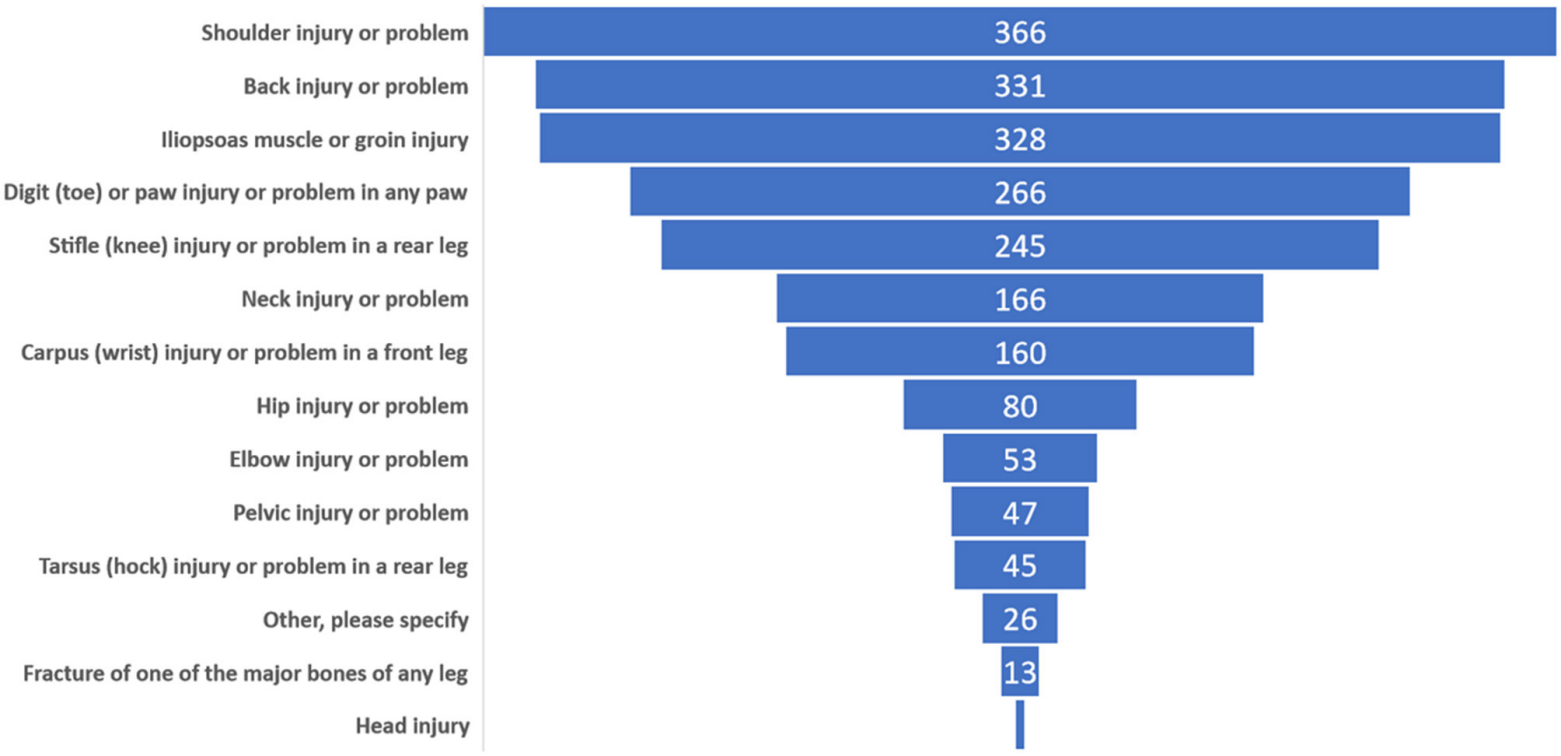
Number of respondents indicating that they had one or more dogs experience an injury at the designated anatomical location which was of sufficient severity to cause the respondent to moderate or stop agility training or competition for a period of time. The number of responses (2,133) was larger than the number of respondents (1,322) because multiple responses were permitted. Injuries described in the category of “Other, please specify,” primarily included tendon or ligament injuries, unspecified or undetermined soft tissue or muscle injuries, lacerations, and eye injuries.

Overall, 1,015 of 1,322 respondents (76.8%) indicated that at least one of their personal dogs had experienced an injury that caused them to moderate or stop agility training. The most frequently reported sites of injury were the shoulder, back, iliopsoas muscle, and digit (Figure 1).

There were 477 respondents (36.1%) who indicated that at least one of their dogs had likely acquired one or more infectious diseases at agility competition or training events through interaction with other dogs (Table 4). The most frequently indicated type of infectious disease was respiratory tract infection (*n* = 345, 26.1%), followed by gastrointestinal tract disease (*n* = 161, 12.2%), and external parasites (*n* = 89, 6.7%). The geographic range of respondents indicating experience with contagious respiratory disease differed from those who indicated no experience with these diseases (*P* = 0.02, Table 5), and a difference in geographic range was also observed among those with and without experience with external parasites (*P* < 0.001). There were lower rates of reported respiratory illness in the Southeast and Western US with higher rates in the Midwest. In contrast, there were higher reports of external parasites in the West and South and lower rates in the Midwest US. A statistically significant difference in geographic range for respondents who indicated experience with gastrointestinal tract disease was not observed (*P* = 0.2). There were too few reports in the “other infectious diseases” group (*n* = 7) to explore differences by geographic region.

**Table 4:** Number (%) of 1,322 respondents indicating whether they believed any of their dogs had acquired various types of infectious diseases as a result of interactions with other dogs at agility training or competition events.

**Infectious disease**	**Number (%) of respondents**
**Respiratory disease**	345 (26.1%)
Kennel cough (infectious tracheobronchitis)	294 (22.2%)
Cough or respiratory disease of unknown type	87 (6.6%)
Canine influenza	11 (0.8%)
**External parasites**	89 (6.7%)
Fleas, ticks, or similar external parasites	89 (6.7%)
**Gastrointestinal diseases**	161 (12.2%)
Diarrhea or vomiting of unknown type	145 (11.0%)
Intestinal parasites of any type, including coccidiosis	28 (2.1%)
Canine coronavirus	4 (0.3%)
**Other infectious diseases**	7 (0.5%)
Leptospirosis	3 (0.2%)
Ocular infection	2 (0.2%)
Rocky Mountain spotted fever	1 (0.1%)
Papilloma virus	1 (0.1%)
**No infectious diseases reported**	845 (63.9%)

Respondents were allowed to select multiple responses.

**Table 5:** Percentage of respondents reporting any history of respiratory disease, external parasites, or gastrointestinal disease following an agility trial.

**First digit of zip code**	**States^∧^**	**Number (%) of respondents reporting disease**	**Adjusted residual**
**Respiratory disease (*****p*** = **0.02)**			
0	CT, MA, ME, NH, NJ, PR, RI, VT	42 (29.6%)	1.00
1	DE, NY, PA	27 (30.0%)	0.87
2	DC, MD, NC, SC, VA, WV	24 (21.1%)	−1.28
3	AL, FL, GA, MS, TN	18 (17.5%)	−2.08^*^
4	IN, KY, MI, OH	41 (33.9%)	2.05^*^
5	IA, MN, MT, ND, SD, WI	34 (23.6%)	−0.72
6	IL, KS, MO, NE	42 (32.8%)	1.82
7	AR, LA, OK, TX	21 (25.9%)	0.04
8	AZ, CO, ID, NM, NV, UT, WY	35 (31.3%)	1.30
9	AK, CA, HI, OR, WA	61 (21.3%)	−2.11^*^
**External parasites (*****p*** = **0.001)**			
0	CT, MA, ME, NH, NJ, PR, RI, VT	11 (7.8%)	0.51
1	DE, NY, PA	5 (5.6%)	−0.46
2	DC, MD, NC, SC, VA, WV	3 (2.6%)	−1.83
3	AL, FL, GA, MS, TN	11 (10.7%)	1.67
4	IN, KY, MI, OH	2 (1.7%)	−2.34^*^
5	IA, MN, MT, ND, SD, WI	4 (2.8%)	−2.00^*^
6	IL, KS, MO, NE	5 (3.9%)	−1.34
7	AR, LA, OK, TX	11 (13.4%)	2.54^*^
8	AZ, CO, ID, NM, NV, UT, WY	7 (6.3%)	−0.21
9	AK, CA, HI, OR, WA	30 (10.5%)	2.84^*^
**Gastrointestinal diseases (*****p*** = **0.2)**			
0	CT, MA, ME, NH, NJ, PR, RI, VT	25 (17.6%)	2.09^*^
1	DE, NY, PA	15 (16.7%)	1.35
2	DC, MD, NC, SC, VA, WV	12 (10.5%)	−0.56
3	AL, FL, GA, MS, TN	13 (12.6%)	0.14
4	IN, KY, MI, OH	16 (13.2%)	0.37
5	IA, MN, MT, ND, SD, WI	13 (9.0%)	−1.23
6	IL, KS, MO, NE	13 (10.2%)	−0.74
7	AR, LA, OK, TX	15 (18.5%)	1.80
8	AZ, CO, ID, NM, NV, UT, WY	10 (8.9%)	−1.10
9	AK, CA, HI, OR, WA	29 (10.1%)	−1.21

*P*-value from the chi square test of any difference by region as well as the adjusted residuals are also shown. Residuals >2 in absolute value are starred (*) indicating greater differences from the overall average. ^∧^States included in each zip code area are indicated by their postal code; state names and names of US territories and armed forces in each zip code area are indicated in Table 1.

There were 1,133 respondents (85.7%) who provided a response to the query related to reasons for retiring a dog from agility competition. The most common reason provided was one or more problems related to advancing age (*n* = 337, 29.7%). Other reasons for retirement included lameness (*n* = 171, 15.1%), behavioral or stress-related issues (*n* = 129, 11.4%), medical issues unrelated to agility (*n* = 113, 10.0%), neck or back pain or problem (*n* = 105, 9.3%), vision problem (*n* = 75, 6.6%), and human factors unrelated to the health of the dog (*n* = 14, 1.2%). Other reasons cited by 189 respondents (16.7%) were varied and might have been more appropriately classified within existing categories. That group also included respondents who had not yet retired a dog from competition (*n* = 18, 1.6%). There were 189 respondents who did not answer this question and it is possible that some of them had not yet retired a dog from competition.

### Research priority rankings

**Figure 2:**
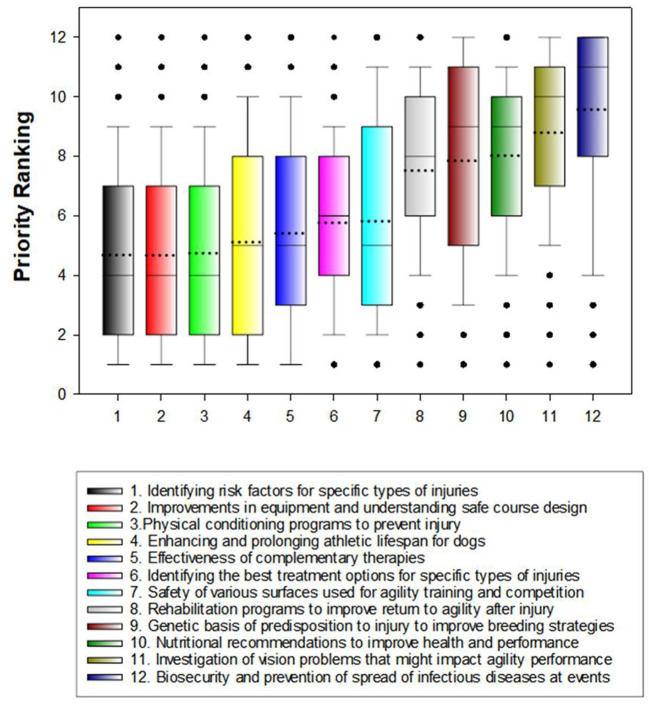
Box and whiskers plots showing priority rankings of agility dog health research areas. Boxes represent 25th and 75th percentiles and whiskers represent 10th and 90th percentiles. The black line inside each box represents the median ranking value for that research area and the dotted black line represents the mean value. Boxes are ordered from highest priority to lowest priority based on mean ranking score. A lower mean ranking indicates a higher priority research area.

Overall median rankings for each broad research area are shown in Figure 2. Differences in median rankings based on preferred organization were small, with no differences statistically significant after correction for multiple comparisons (Table 6).

**Table 6:** Median (IQR) priority rankings for each research topic as provided by all respondents or by specified subsets of respondents.

**Research topic**	**Overall ranking**	**All respondents**	**Preferred agility organization**						
			**AKC (*****n*** = **724)**	**ASCA (*****n*** = **43)**	**CPE (*****n*** = **196)**	**NADAC (*****n*** = **68)**	**UKI (*****n*** = **67)**	**USDAA (*****n*** = **180)**	***P*** **-value** ^*^
Identifying risk factors for specific types of injuries	1	4 (2–7)	5 (2–7)	5 (2–7)	4.5 (3–7)	4 (2–7)	4 (3–6)	4 (2–7)	0.34
Improvements in equipment and understanding safe course design	1	4 (2–7)	4 (2–7)	4 (3–8)	4 (2–7)	3 (2–6)	3 (2–7)	4 (2–7)	0.18
Physical conditioning programs to prevent injury	1	4 (2–7)	4 (2–7)	5 (3–6)	4 (2–7)	4 (2–6)	4 (3–7)	5 (2–7)	0.80
Enhancing and prolonging the athletic lifespan for dogs	4	5 (2–8)	5 (2–8)	4 (2–8)	4 (2–8)	5 (2–8)	5 (3–8)	4 (2–7)	0.32
Effectiveness of complementary therapies such as chiropractic, acupuncture, massage, magnetic, etc.	4	5 (3–8)	5 (2–9)	4 (2–7)	5 (3–8)	6 (4–8)	6 (3–9)	5 (3–7.8)	0.32
Safety of various surfaces used for agility training and competition	4	5 (3–9)	5 (3–9)	8 (4–10)	5 (2–8)	5 (2–8)	5 (2–8)	5 (3–9)	0.13
Identifying the best treatment options for specific types of injuries	7	6 (4–8)	6 (4–8)	6 (3–7)	5 (4–7)	6 (4–8)	6 (4–8)	6 (4–8)	0.50
Rehabilitation programs to improve return to agility after injury	8	8 (6–10)	7 (6–9)	7 (6–10)	8 (6–10)	8 (5–10)	8 (5–10)	8 (6–10)	0.39
Genetic basis of predisposition to injury to improve breeding strategies	9	9 (5–11)	8 (5–11)	7 (4–10)	9 (6–11)	10 (7–11)	7 (4–10)	9 (6–11)	0.009
Nutritional recommendations to improve health and performance	9	9 (7–10)	9 (6–11)	9 (6–11)	8 (5–10)	9 (6–11)	9 (7–10)	8.5 (6–10)	0.041
Investigation of eye or vision problems that might impact agility (e.g., early takeoff syndrome)	11	10 (7–11)	10 (7–11)	10 (8–11)	10 (7–11)	10 (8–11)	9 (6–11)	9 (7–10)	0.017
Biosecurity and prevention of spread of infectious diseases at events	12	11 (8–12)	11 (8–12)	11 (8–12)	11 (7–12)	10 (6.3–12)	11 (8–12)	12 (10–12)	0.007

For each individual research topic, rankings by competitors specifying a preferred agility organization were compared using a Kruskal-Wallis analysis of variance on ranks. ^*^No *P*-values were significant after Holm adjustment for multiple comparisons (12 tests). AKC, American Kennel Club; ASCA, Australian Shepherd Club of America; CPE, Canine Performance Events; NADAC, North American Dog Agility Council; UKI, United Kingdom Agility International; USDAA, United States Dog Agility Association.

Veterinarians and credentialed veterinary technicians competing in agility (*n* = 94, 7.3%) ranked effectiveness of complementary therapies as a lower research priority area than non-veterinary professionals (median ranking 7 vs. 5, *P* < 0.001) but no other differences were statistically significant after correction for multiple comparisons (Table 7). Comparisons of rankings for competitors who indicated that they had previously competed in at least one national championship event and those that did not revealed a difference only in ranking for eye or vision problems in which national championship competitors ranked this issue slightly higher (median ranking 9 vs. 10, *P* = 0.003; Table 7).

**Table 7:** Median (IQR) priority rankings for each research topic as provided by all respondents or by specified subsets of respondents.

**Research topic**	**Overall ranking**	**All respondents**	**Veterinarian or credentialed veterinary technician**			**Competed at a national championship event**		
			**Yes (*****n*** = **94)**	**No (*****n*** = **1,200)**	***P*** **-value** ^*^	**Yes (*****n*** = **645)**	**No (*****n*** = **648)**	***P*** **-value** ^*^
Identifying risk factors for specific types of injuries	1	4 (2–7)	4 (3–7)	4 (2–7)	0.50	4 (2–7)	4 (2–7)	0.74
Improvements in equipment and understanding safe course design	1	4 (2–7)	4 (2–8)	4 (2–7)	0.29	4 (2–7)	4 (2–7)	0.72
Physical conditioning programs to prevent injury	1	4 (2–7)	4 (2–6)	4 (2–7)	0.79	4 (2–6)	4 (2–7)	0.91
Enhancing and prolonging the athletic lifespan for dogs	4	5 (2–8)	4 (2–6)	5 (2–8)	0.055	5 (2–8)	4 (2–8)	0.26
Effectiveness of complementary therapies such as chiropractic, acupuncture, massage, magnetic, etc.	4	5 (3–8)	7 (4–10)	5 (3–8)	< 0.001^*^	5 (3–8)	5 (3–8)	0.23
Safety of various surfaces used for agility training and competition	4	5 (3–9)	6 (3–9)	5 (3–9)	1.0	5 (2–8)	6 (3–9)	0.075
Identifying the best treatment options for specific types of injuries	7	6 (4–8)	6 (4–8)	6 (4–8)	0.99	6 (4–8)	6 (4–8)	0.63
Rehabilitation programs to improve return to agility after injury	8	8 (6–10)	7 (5–9)	8 (6–10)	0.015	8 (6–10)	8 (6–9)	0.11
Genetic basis of predisposition to injury to improve breeding strategies	9	9 (5–11)	8 (4–10)	9 (6–11)	0.043	9 (6–11)	9 (5–11)	0.60
Nutritional recommendations to improve health and performance	9	9 (6–10)	10 (7–11)	9 (6–10)	0.007	9 (6–10)	9 (6–11)	0.41
Investigation of eye or vision problems that might impact agility (e.g., early takeoff syndrome)	11	10 (7–11)	9 (6–10)	10 (7–11)	0.023	9 (7–11)	10 (8–11)	0.003^*^
Biosecurity and prevention of spread of infectious diseases at events	12	11 (8–12)	12 (9–12)	11 (8–12)	0.010	11 (9–12)	11 (8–12)	0.08

Responses from veterinarians and credentialed veterinary technicians were compared to responses from non-veterinary professionals using a Mann-Whitney rank sum test. Responses from individuals who had competed in at least one national championship event of any venue in the past 5 years were similarly compared to responses from individuals who had not competed in a national championship event. ^*^Asterisks indicate a significant difference after Holm adjustment for multiple comparisons (12 tests within each category).

**Figure 3:**
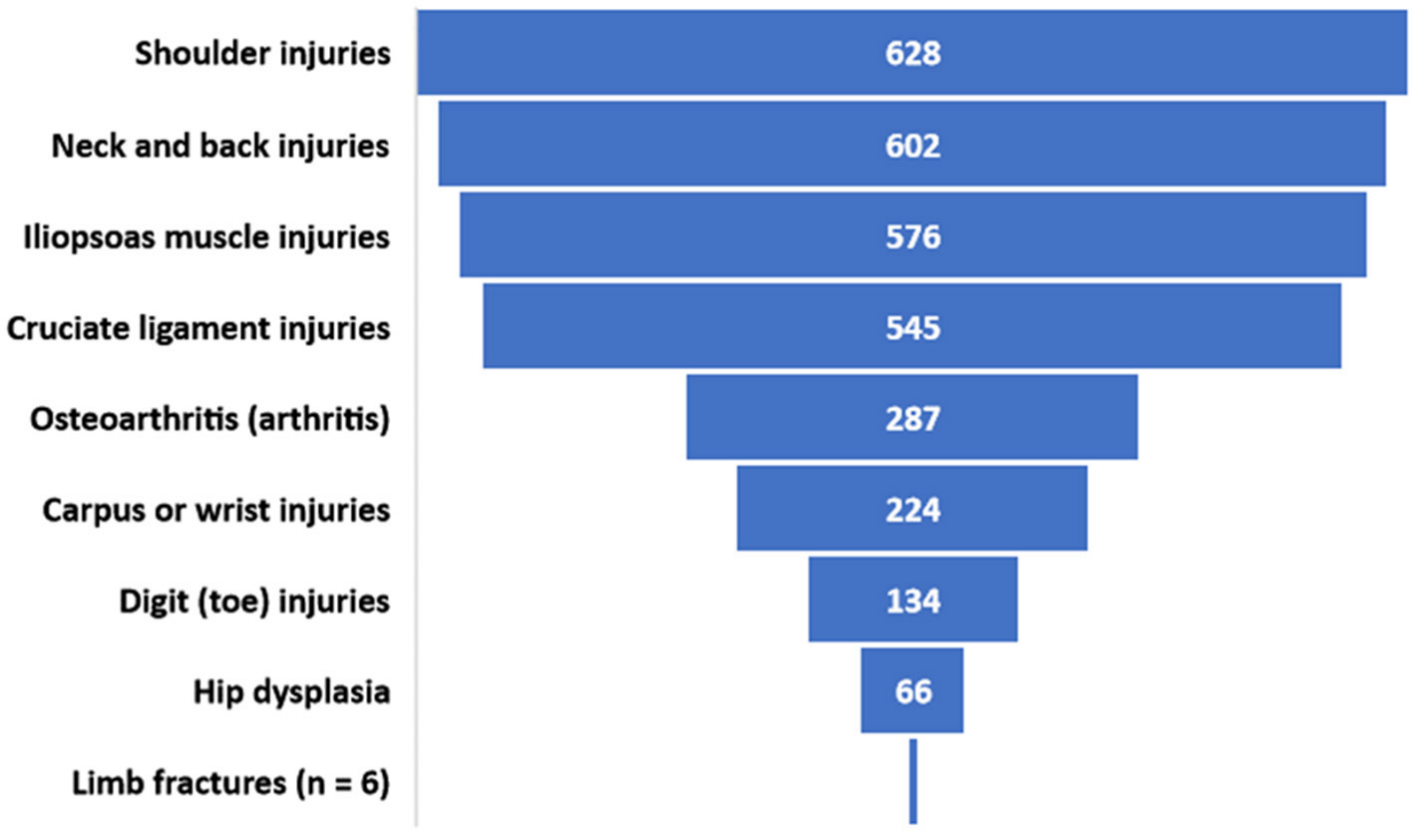
Number of respondents who included each type of musculoskeletal injury or problem in their designation of up to 3 priority research recommendations.

Respondents selected up to three topics of highest priority (unranked) for musculoskeletal injury research related to agility dogs. Responses from 39 individuals who indicated more than 3 injury types and 9 individuals who indicated no types of injury were excluded from analysis. The final data set for analysis of this question included responses from 1,277 individuals, each of whom could indicate up to 3 areas of research. Musculoskeletal injury research priorities are indicated in Figure 3.

Respondents were asked to identify in free text responses any other research areas that they considered important. The most frequent responses that did not relate to a previously listed research area were related to behavior, stress, and mental well-being of the dogs.

## Discussion

This survey of US agility competitors sought to better understand adverse health events encountered as a result of sport participation and canine health research priorities of competitors. The results demonstrate that competitor experiences and research priorities are remarkably uniform regardless of their preferred agility organization. There is a strong desire for information that will advance the safety of the sport through increased understanding of risk factors for injury and injury prevention strategies which might include improvements in equipment, understanding safe course design, development of physical conditioning programs for dogs, and research regarding safety of various surfaces used for training and competition.

It is challenging to determine whether the respondents to this questionnaire are appropriately representative of all agility participants in the US. The current study did not ascertain gender, age, or educational status of respondents except as related to veterinary medical training (veterinarian or credentialed veterinary technician). There have been several internet-based surveys of agility participants, however, that have produced consistent demographic descriptors of sport participants (7, 13, 14, 18–22, 24). In the largest and most recent of these surveys (18), respondents were predominantly female (>90%) and over the age of 45 years (>50%). More than half had a 4-year college degree or higher (graduate, professional) level of education.

The percentage of North American respondents who were veterinarians or veterinary technicians in the survey by Sundby and colleagues was very similar to this current report (6.7 and 7.1%, respectively) (18). The number of years respondents had been participating in agility and the percentage of respondents who had competed at a national championship event (54.4 and 49.8%, respectively) were also similar (18). It was not possible to determine whether the geographic distribution of responses or the distribution of responses related to preferred agility organization were representative of the US agility population, however, because there are no known data for comparison. Given the sample size calculations and the similarity of experience and demographics between this report and other internet-based studies, it is reasonable to conclude that the information obtained was appropriately representative of US agility participants. A risk for selection and response bias remains with internet-based survey research. Because previous reports used similar sampling methods, there is a risk that all surveys represent primarily the opinions of the most engaged agility competitors.

Respondents were asked to report adverse health events, defined as infectious diseases or injuries, that had occurred in one or more of their dogs as a result of participation in agility activities. Approximately 1/3 of respondents indicated that one or more dogs had acquired an infectious disease as a result of agility activities, with respiratory disease reported by 26% of respondents. It is not possible to ascertain whether these diagnoses are accurate, whether the respiratory signs which were observed were truly the result of an infectious disease, or whether the disease had actually been acquired as a result of participation in an agility activity. The high number of reports, however, are consistent with observations that more than 10% of asymptomatic client-owned dogs and almost 50% of asymptomatic dogs presenting to US animal shelters have one or more canine infectious respiratory pathogens detectable on ocular or oronasal swabs (34, 35). The apparent differences in incidence of specific types of infectious diseases by regions are intriguing and may be related to weather or differences in the types of agility venues that predominate (indoor or outdoor, ventilation of facilities, etc.), but results should be interpreted with caution given the lower number of respondents in some areas.

High-profile infectious disease outbreaks have occurred in association with equine events with devastating consequences for the industry across broad geographic areas (36–38). There is risk for similar large-scale infectious disease outbreaks at dog events given the large number of participants who travel to national championship events. Veterinary textbooks related to canine infectious diseases do not include comprehensive discussion of canine biosecurity, except in kennel environments (39), although extensive disease prevention guidelines are available (28). In contrast, similar textbooks for equine infectious diseases include detailed discussion of biosecurity and control of disease outbreaks related to horse events (40) and major veterinary and equine sport organizations publish a variety of infectious disease control guidelines for event organizers (41–44). Understanding the movement patterns of dogs, biosecurity practices, vaccination status, types of competition venues, and other factors that might influence the spread of disease, especially respiratory pathogens, is very important in determining recommendations regarding best practices for event biosecurity. Despite the high incidence of competitor reports of infectious diseases in agility dogs, neither competitors nor veterinarians considered biosecurity practices to be a high priority area for future research. This low prioritization would likely change if there were a widespread infectious disease outbreak among agility dogs competing at a large national event.

Several previous reports have focused on the anatomic distribution of injuries in agility dogs (13, 22, 24). Results of those studies and the current study are remarkably similar, with the identification of shoulder, back, iliopsoas muscle, and digit injuries as the most common sites of injury. Despite this consistent finding, there is little research that has specifically investigated the nature, cause, treatment, or prognosis related to shoulder, back, or iliopsoas injuries in agility dogs (23). The data must also be interpreted with some caution because all reports have relied exclusively on owner-provided information regarding the anatomic location or diagnosis of the injury rather than review of veterinary medical records. Not surprisingly, because of the highest rate of injury, respondents ranked the shoulder, back, and iliopsoas injuries as the highest priority for future research on musculoskeletal injuries.

This is the first report of reasons for retirement of dogs from agility competition, although there have been descriptions for police dogs (45), working farm dogs (46), assistance dogs (47), and gundogs (48). The most frequently cited reasons for retirement from agility competition were advancing age, lameness, behavioral or stress-related issues, medical issues unrelated to agility, and neck or back pain or problem. Human factors unrelated to the health of the dog were cited as a reason for retirement from competition by only 1.2% of respondents. For working police dogs, degenerative musculoskeletal disease was cited as a reason for retirement for 69% of dogs (45). When euthanasia and retirement were considered together, back or spinal problems were cited for 27% of dogs with a high proportion believed to involve the lumbosacral joint (45). A prospective longitudinal study of 126 working farm dogs in New Zealand revealed that lameness was a major risk factor for loss from the work force, but specific diagnoses or causes of lameness were not reported (46). The majority of assistance dogs in one study (6,465/7,686; 84%) worked until scheduled retirement age of 8.5 years. The most common reasons for early retirement were musculoskeletal conditions (47). For gundogs, the most common reasons for retirement were lameness (25.5%), old age (23.7%), and deafness (7.8%) (48).

Behavioral or stress-related conditions were cited as a primary reason for retirement of an agility dog by > 10% of respondents. This finding was unique as compared to other types of working or sport dogs. Some possible causes of behavior or stress-related impediments to agility include unrecognized physical pain that affects behavior (49), training by amateur dog handlers with assistance from coaches or instructors with variable levels of formal certification as dog trainers, synchronization with stress levels in the dogs' owners (50), or these retirements may reflect a direct relationship with the inherent stress of the sport (4) or be related to characteristics of the breeds that predominate in the sport (i.e., border collies, Australian shepherds, Shetland sheepdogs, and mixed breeds) (13, 14, 20, 21). Alternatively, other types of working dogs (e.g., military and police dogs) may be more intensely screened for temperament prior to training with removal of dogs displaying significant social anxiety or similar behavioral challenges. Understanding the causes for behavior and stress-related retirements from agility appears worthy of further investigation. This research area was not included for ranking in the list of research priority areas in this study, but related topics (stress, behavior, mental well-being) were mentioned by multiple respondents when asked to specify additional types of research that are needed.

Agility dog health research priority rankings are very similar regardless of respondents' preferred agility organization or level of involvement in the sport as reflected by participation in one or more national championship events. Overall, agility competitors placed the highest priority on research that would decrease risk of injury in their dogs: identifying risk factors for specific types of injury, improvements in equipment and understand of safe course design, and physical conditioning programs to prevent injury. While there is some data available about risk factors for digit injuries (20) and cranial cruciate ligament rupture (14) in agility dogs, these are not the most frequent or highest priority musculoskeletal problems identified in this study. Although there have been a few reports related to performance by agility dogs of specific agility obstacles such as the A-frame (2, 3, 5), jumps (5, 12, 17, 51–53), and weave poles (6), there are few reports related to course design or competition surface and their effects on likelihood of injury (25, 54). Similarly, there are no reports of effects of specific physical conditioning programs to prevent injury, although one study reports an association between weekly core strengthening and balance exercises and decreased risk of cranial cruciate ligament rupture (14).

After correction for multiple comparisons, the only difference in research rankings between veterinary professionals and other competitors was that veterinary professionals considered investigations of the effectiveness of complementary therapies to be of lesser importance. The interpretation of this finding is complicated by the comparatively low number of veterinary professionals (*n* = 94) included in the analysis and additional research is required to better understand this finding. There is likely a relationship between the lesser importance placed on complementary therapies by veterinary professionals and the strong emphasis on Western medicine in most veterinary educational curricula in the US.

Agility competitors prioritize research that is likely to contribute to strategies that will prevent injury to their dogs. These priorities are uniform regardless of the preferred agility organization and experience level of the competitor. This information should serve as a call to all stakeholders in the diverse sport of agility to communicate and collaborate with a common goal of improved health and well-being for the canine athlete in the team sport of agility. Collaboration in developing research strategies, identifying funding support, and prospectively collecting data is required.

## Data availability statement

The raw data supporting the conclusions of this article will be made available by the authors, without undue reservation.

## Ethics statement

The studies involving human participants were reviewed and approved by the Washington State University Institutional Review Board. Written informed consent for participation was not required for this study in accordance with the national legislation and the institutional requirements.

## Author contributions

DS, DM-L, DM, and MM participated in all aspects of study design, data collection, data analysis, and writing of the manuscript. AP participated in data collection, data analysis, and writing of the manuscript. AS participated in data analysis and writing the manuscript. All authors contributed to the article and approved the submitted version.
