# Supplementary material for: Adverse health events and recommended health research priorities in agility dogs as reported by dog owners
Source: Front Vet Sci. 2023 Mar 13;10:1127632. doi: 10.3389/fvets.2023.1127632 (PMC10040666; doi:10.3389/fvets.2023.1127632)
Supplement: Supplementary file 1 [file Data_Sheet_1.pdf]

Agility Dog Health Research Priorities

Introduction

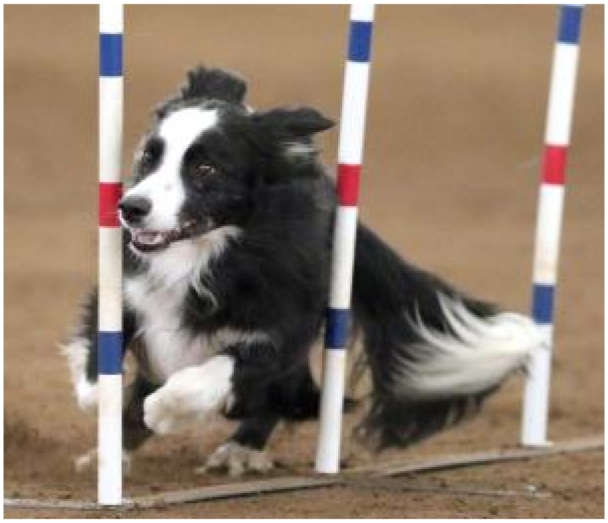

Introduction

Purpose: Dogs participating in agility may have some health needs or disease risks that differ from those of pet dogs that are not athletically active. Our goals are:

(1) to identify the health concerns of most significance to individuals participating with their dogs in agility events; and,
(2) to determine how agility handlers prefer to obtain veterinary health care information.

The investigators will use the information derived from this survey to influence prioritization of research initiatives related to the health and well-being of dogs participating in agility events. Our goals are to stimulate discussions with veterinary and canine organizations, including agility organizations, in an effort to develop strategic partnerships to advance canine agility health research and disseminate important health information to competitors.

Eligibility: Any individual at least 18 years of age who is currently involved in the sport of dog agility in the United States with one or more dogs is eligible to participate in this survey.

Participation: Participation in this study is completely voluntary and

anonymous. No details about yourself or your dogs will be collected beyond what you choose to provide. You may quit the survey at any time.

Estimated Time: This survey takes approximately 15 minutes to complete. There will be questions about your agility experiences, sources of veterinary care and veterinary information, and opportunities to indicate which health concerns related to agility dogs are most important to you.

Who: The research team for this project includes Dr. Debra Sellon (Professor of Equine Medicine at Washington State University), Dr. Denis Marcellin-Little (Professor of Small Animal Orthopedic Surgery at University of California, Davis), Dr. Molly McCue (Professor of Equine Internal Medicine at University of Minnesota), and Dr. Dianne McFarlane (Professor of Equine Research at Oklahoma State University).

Ethics: This study was deemed exempt from review by the Institutional Review Board (IRB) at Washington State University.

If you have any questions, comments, or concerns, please contact Dr. Debra Sellon at canineagilityresearch@wsu.edu.

Reside in US

Are you 18 years of age or older?

- ◯ Yes
- ◯ No

Do you reside in the United States?

- ◯ Yes

- ◯ No

Have you competed in at least one agility trial with one or more dogs in the previous 6 months?

- ◯ Yes
- ◯ No

Experiences with Health Issues in Agility Dogs

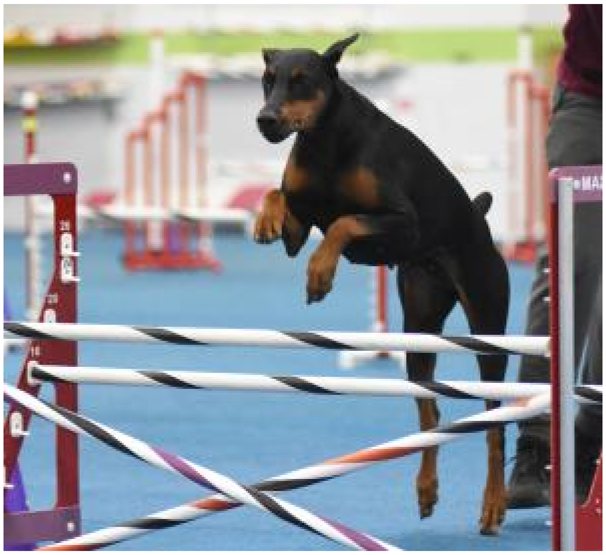

In this section we will ask questions to determine how various canine health issues have affected you and your agility dogs. This will help us learn more about the frequency of specific types of health problems among the general agility dog population.

This question seeks to identify the most common anatomical sites for ***injury*** in agility dogs. Please indicate whether any of your personal dogs have experienced an ***injury*** related to the anatomical body part listed. Include only those injuries which caused you to moderate or stop agility training or competition. Choose all that apply.

- ☐ Head injury
- ☐ Neck injury or problem
- ☐ Back injury or problem
- ☐ Shoulder injury or problem
- ☐ Elbow injury or problem

- [ ] Carpus (wrist) injury or problem in a front leg
- [ ] Digit (toe) or paw injury or problem in any paw
- [ ] Pelvic injury or problem
- [ ] Hip injury or problem
- [ ] Iliopsoas or groin injury
- [ ] Stifle (knee) injury or problem in a rear leg
- [ ] Tarsus (hock) injury or problem in a rear leg
- [ ] Fracture of one of the major bones of any leg
- [ ] ________ Other, please specify

Do you believe that any of your dogs have acquired the following infectious diseases as a result of interactions with other dogs at agility training or competition events? Choose all that apply.

- [ ] Canine coronavirus
- [ ] Canine distemper
- [ ] Canine hepatitis virus
- [ ] Canine influenza
- [ ] Cough or respiratory disease of unknown type
- [ ] Diarrhea or vomiting of unknown type
- [ ] Fleas, ticks, or similar external parasites
- [ ] Intestinal parasites of any type, including coccidiosis
- [ ] Kennel cough (infectious tracheobronchitis)
- [ ] Leptospirosis
- [ ] Parvovirus
- [ ] ________ Other, please specify

Consider the most recent dog which you have retired from agility competition. Which one of the following reasons best explains your decision to retire that

dog?

- ◯ Behavioral or stress-related issues associated with travel or competition
- ◯ Human factors (dog was fine, human had reasons to quit competing)
- ◯ Lameness - pain or injury associated with legs, hips, feet
- ◯ Medical issues unrelated to agility such as diabetes, autoimmune disease, cancer, etc.
- ◯ Neck or back pain or problem such as disc problems, spondylosis, etc.
- ◯ One or more problems related to advancing age
- ◯ Vision problem
- ◯ [ ] Other, please specify

Research Priorities

In this section we want you to provide us with your opinions on specific research topics that you believe would be of most benefit to agility dog health and well-being.

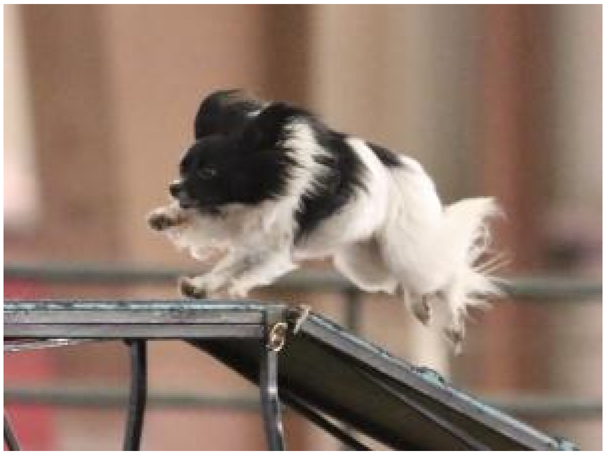

Which of the following general research areas do you think are most important for advancing and protecting the health of dogs participating in agility training and competitions? ***Please rank the options from 1 (most important) to 11 (least important) by dragging the option to the appropriate position in the list.***

Biosecurity and prevention of spread of infectious diseases at events

Effectiveness of complementary therapies such as chiropractic, acupuncture, massage, magnetic, etc.

Enhancing and prolonging athletic lifespan for dogs

Genetic basis of predisposition to injury to improve breeding strategies

Identifying risk factors for specific types of injuries

Identifying the best treatment options for specific types of injuries

Improvements in equipment and understanding safe course design

Investigation of eye or vision problems that might impact agility performance (e.g. early takeoff syndrome)

Nutritional recommendations to improve health and performance

Physical conditioning programs to prevent injury

Rehabilitation programs to improve return to agility after injury

Safety of various surfaces used for agility training and competition

Which of the following individual musculoskeletal injuries do you think are most important to target in future research initiatives to improve health of agility dogs? Please click to indicate the ***three topics*** you think should have the highest priority. (If you want to change your selection after clicking, click again and you will deselect that option and can then choose another.)

- ☐ Carpus or wrist injuries
- ☐ Cruciate ligament injuries
- ☐ Digit (toe) injuries
- ☐ Hip dysplasia
- ☐ Iliopsoas injuries
- ☐ Limb fractures
- ☐ Neck and back injuries
- ☐ Osteoarthritis (arthritis)

- ☐ Shoulder injuries
- ☐ Soft tissue injuries

What other health concerns do you believe should be prioritized in future research initiatives for agility dogs?

Sources of Agility Dog Health Information

In this section we seek to learn more about how you prefer to receive new health care information related to agility dogs and which information sources you find most trustworthy.

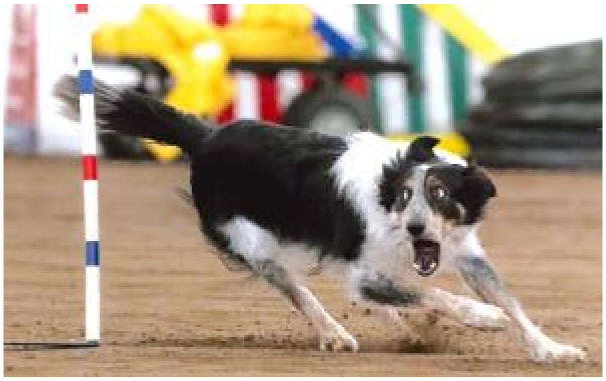

How knowledgeable are you about the types of injuries/health problems that are common in agility dogs?

Not at all knowledgeable — Extremely knowledgeable

0 ○ 1 ○ 2 ○ 3 ○ 4 ○ 5 ○ 6 ○ 7 ○ 8 ○ 9 ○ 10 ○

How knowledgeable is your regular primary care veterinarian about the sport of agility and the types of injuries/health problems that are common in agility dogs?

Not at all knowledgeable — Extremely knowledgeable

0 ○ 1 ○ 2 ○ 3 ○ 4 ○ 5 ○ 6 ○ 7 ○ 8 ○ 9 ○ 10 ○

Rate the level of trust you have for the following sources of health-related information for your agility dogs.

	Generally trustworthy	Not generally trustworthy
Agility or dog related websites (nonveterinary)	○	○
Books, magazines, or similar print information for dog owners	○	○
Information from breed organizations	○	○
Information from universities, veterinary schools	○	○
Instructor/coach - personal communications	○	○
Online seminars, workshops, and courses	○	○
Other agility competitors - personal communications	○	○
Peer-reviewed scientific articles	○	○
Personal knowledge	○	○
Pet food manufacturer representatives	○	○
Pet store personnel	○	○
Social media posts from individuals: Facebook, Twitter, etc.	○	○
Traditional news sources: radio, TV, newspaper	○	○
Veterinarian who provides regular care for my dogs	○	○
Veterinarian with special training or expertise in sports medicine	○	○

From which of the following sources are you ***most likely*** to ***first learn*** about new information related to agility dog health - such as information about a new or emerging disease or a new treatment for a specific type of injury? ***Please choose your top 3 sources!***

☐ Direct communication with your agility instructor or coach

- ☐ Direct communication with other competitors and friends
- ☐ Direct communication with a veterinarian
- ☐ Electronic newsletter with a focus on agility dogs
- ☐ Information provided by a local agility or dog-training club
- ☐ On-line seminar, workshop, or similar event
- ☐ Social media posts shared by friends - such as Facebook, Twitter, or similar
- ☐ Traditional general news source such as radio, television, newspapers
- ☐ Web site primarily for agility dog topics, please specify which one ______
- ☐ Website for commercial products or services, please specify which one ______
- ☐ Web site for general dog health information, please specify which one ______
- ☐ Web site for professional veterinarian or veterinary association, please specify ______ which one

Is there another source of information, other than those listed in the questions above, that you use and trust for information related to the health of your agility dogs?

______

Background and Experiences

We will conclude by asking a few questions about your experiences with dogs and your experiences in canine agility.

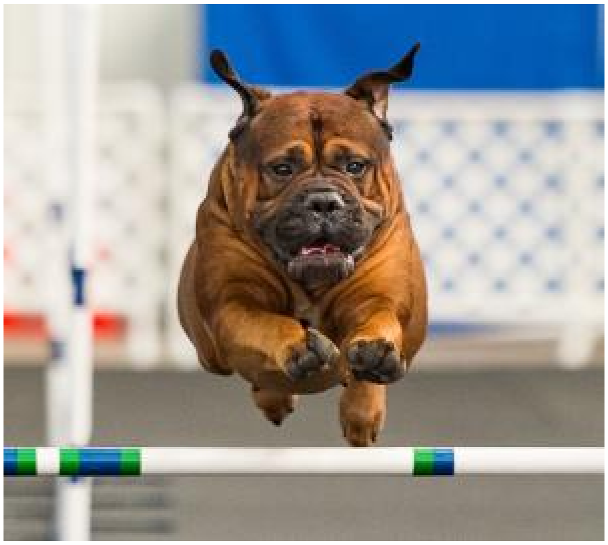

How many dogs do you currently own that actively compete in agility? (Do not include dogs in training that are not yet competing or dogs that have retired from competition.)

How many dogs have you owned in your lifetime which you trained and/or competed with in agility?

For how many years have you been actively involved in canine agility training and competition?

On average, how many ***total days per year*** do you compete in agility?

In the past 10 years, have you been involved with dog breeding as the owner of either the dam or sire of a litter of puppies?

- ◯ Yes
- ◯ No

In the past 5 years, in which of the following agility venues have you competed? Check all that apply.

- ☐ American Kennel Club (AKC)
- ☐ Australian Shepherd Club of America (ASCA)
- ☐ Canine Performance Events (CPE)
- ☐ Dogs on Course in North America (DOCNA)
- ☐ North American Dog Agility Council (NADAC)
- ☐ Teacup Dogs Agility Association (TDAA)
- ☐ United States Dog Agility Association (USDAA)
- ☐ United Kingdom Agility International (UKI)
- ☐ United Kennel Club (UKC)
- ☐ ______ Other, please specify

In the past 5 years, in which one of the following agility venues have you competed most frequently? Check only one.

- ◯ American Kennel Club (AKC)
- ◯ Australian Shepherd Club of America (ASCA)
- ◯ Canine Performance Events (CPE)
- ◯ Dogs on Course in North America (DOCNA)
- ◯ North American Dog Agility Council (NADAC)
- ◯ Teacup Dogs Agility Association (TDAA)
- ◯ United States Dog Agility Association (USDAA)
- ◯ United Kingdom Agility International (UKI)
- ◯ United Kennel Club (UKC)
- ◯ ______ Other, please specify

In the past 5 years, have you competed with one or more dogs in a national championship agility event (any venue)?

- ◯ Yes
- ◯ No

Which of the following best describe your current participation in the sport of dog agility? Choose all that apply.

- ☐ Active competitor and trainer with my personal dogs
- ☐ Active competitor with dogs owned by other individuals
- ☐ Agility judge
- ☐ Agility trial secretary or similar activity
- ☐ Currently practicing or retired credentialed veterinary technician
- ☐ Currently practicing or retired veterinarian
- ☐ Dog breeder
- ☐ Instructor for other agility handlers and their dogs (paid for services)
- ☐ Member of the leadership group of an agility organization such as AKC or NADAC
- ☐ Owner or manager of an agility or dog-related retail business
- ☐ Owner or manager of a dog sports venue
- ☐ Training a dog but not currently competing
- ☐ ________ Other, please specify

What is the ***first digit*** of the ***zip code*** for your primary mailing address?

(This information will help us determine whether the information we receive differs based on the region of the US in which you live.)

- ◯ 0

- ◯ 1
- ◯ 2
- ◯ 3
- ◯ 4
- ◯ 5
- ◯ 6
- ◯ 7
- ◯ 8
- ◯ 9

Powered by Qualtrics
